# Analysis of Research Hotspots and Development Trends in the Diagnosis of Lung Diseases Using Low-Dose CT Based on Bibliometrics

**DOI:** 10.2174/0115734056402094250530075121

**Published:** 2025-06-05

**Authors:** Xiaoyu Chen, Xi Liu, Yang Jiang, Yiming Chen, Dechuan Zhang, Longling Fan

**Affiliations:** 1 College of Faculty of Science, Kunming University of Science and Technology, Kunming, China; 2 Department of Radiology, Chongqing Hospital of Traditional Chinese Medicine, Chongqing, China

**Keywords:** Low-dose CT, Lung cancer screening, Artificial intelligence, Bibliometrics, CiteSpace, Cancer

## Abstract

**Background:**

Lung cancer is one of the main threats to global health, among lung diseases. Low-Dose Computed Tomography (LDCT) provides significant benefits for its screening but also brings new diagnostic challenges that require close attention.

**Methods:**

By searching the Web of Science core collection, we selected articles and reviews published in English between 2005 and June 2024 on topics such as “Low-dose”, “CT image”, and “Lung”. These literatures were analyzed by bibliometric method, and CiteSpace software was used to explore the cooperation between countries, the cooperative relationship between authors, highly cited literature, and the distribution of keywords to reveal the research hotspots and trends in this field.

**Results:**

The number of LDCT research articles show a trend of continuous growth between 2019 and 2022. The United States is at the forefront of research in this field, with a centrality of 0.31; China has also rapidly conducted research with a centrality of 0.26. The authors' co-occurrence map shows that research teams in this field are highly cooperative, and their research questions are closely related. The analysis of highly cited literature and keywords confirmed the significant advantages of LDCT in lung cancer screening, which can help reduce the mortality of lung cancer patients and improve the prognosis. “Lung cancer” and “CT” have always been high-frequency keywords, while “image quality” and “low dose CT” have become new hot keywords, indicating that LDCT using deep learning techniques has become a hot topic in early lung cancer research.

**Discussion:**

The study revealed that advancements in CT technology have driven in-depth research from application challenges to image processing, with the research trajectory evolving from technical improvements to health risk assessments and subsequently to AI-assisted diagnosis. Currently, the research focus has shifted toward integrating deep learning with LDCT technology to address complex diagnostic challenges. The study also presents global research trends and geographical distributions of LDCT technology, along with the influence of key research institutions and authors. The comprehensive analysis aims to promote the development and application of LDCT technology in pulmonary disease diagnosis and enhance diagnostic accuracy and patient management efficiency.

**Conclusion:**

The future will focus on LDCT reconstruction algorithms to balance image noise and radiation dose. AI-assisted multimodal imaging supports remote diagnosis and personalized health management by providing dynamic analysis, risk assessment, and follow-up recommendations to support early diagnosis.

## 1 INTRODUCTION

According to the National Comprehensive Cancer Network (NCCN) guidelines [1], lung cancer is one of the most prevalent and deadly cancers worldwide. Its early clinical symptoms and signs are subtle, making diagnosis more difficult. It usually occurs in late stages, leading to poor prognosis and low survival rates. The International Institute for Research on Cancer (IARC) reports that in 2022 [2], there will be nearly 20 million new cancer patients globally, with 9.7 million cancer-related deaths. Lung cancer accounts for 12.4% of all new cancer cases, reaching 2.5 million people. In China, the National Cancer Center statistics showed that there are approximately 780,000 new lung cancer cases annually, accounting for about 38% of the worlds total [3]. The National Cancer Institute notes that the five-year survival rate for stage I lung cancer is approximately 57%, while stage IV is only 5% [4]. The survival rate for early-stage lung cancer is significantly higher than that of late-stage cancer. Researchers have emphasized the importance of early screening and treatment for lung cancer to enhance patient prognosis, prolong life expectancy, and improve overall survival rates [5]. In the early stages of lung cancer development, the tumor cells will appear as lung nodules. Most lung nodules are benign, however, approximately 5%-15% are malignant [6]. Therefore, early detection and accurate evaluation of lung nodules are effective for early detection, diagnosis, and lung cancer treatment.

Computed Tomography (CT) has become an important tool for early detection, diagnosis, and monitoring of diseases, especially those affecting the lungs, and is widely used in clinical settings [7]. However, conventional CT imaging techniques often involve high levels of radiation exposure, and repeated CT scans may increase the risk of radiation-induced illnesses and may worsen the development of the disease [8]. Due to concerns about the harm of excessive CT radiation dose to the human body, researchers are studying techniques to reduce radiation dose while maintaining the accuracy of disease detection and diagnosis. Low-Dose Computed Tomography (LDCT) technology can reduce radiation by decreasing the X-ray during scanning, optimizing scanning parameters, and using complex image-reconstruction algorithms such as iterative reconstruction [9]. With the progress of computer technology, methods containing artificial intelligence have gradually been used to solve image problems. For example, deep learning techniques like CNN [10] can be applied to image denoising, enhancement and preprocessing, feature extraction, and object detection. This method not only reduces the cost of repeated experiments but also enhances detection efficiency, sensitivity, and versatility. Therefore, further research on LDCT technology to reduce radiation dose and improve diagnostic accuracy while ensuring image quality is of great significance.

At present, LDCT technology has shown significant advantages in the field of lung cancer screening. Numerous recent studies have confirmed [11, 12] that using LDCT for routine early screening of high-risk lung cancer patients can significantly improve the accuracy of early diagnosis, effectively improve patient prognosis, and reduce mortality. The research on LDCT technology not only promotes innovation within medical imaging but also has an intricate relationship with multi-disciplinary fields such as radiobiology, radiation dosimetry, and image processing and analysis [13]. In-depth research on LDCT imaging technology can not only improve the accuracy of disease diagnosis but also provide new research ideas and methods for the development of related disciplines. In previous research studies [14, 15], researchers only discussed issues such as CT scanning technology or lung diseases. This study focuses on the application of low-dose CT technology in the diagnosis of lung diseases. A comprehensive bibliometric analysis was conducted on the hotspots and trends in the field of LDCT diagnosis of lung diseases from three dimensions: research objects, research questions, and research methods, revealing the development direction and providing a reference for subsequent research.

## 2 MATERIALS AND METHODS

### 2.1 Research Methods

The primary research method used in this paper is bibliometrics, which is a technique that uses mathematical and statistical approaches to dissect literature and its citation patterns [16]. This method considers various metrics such as the volume of the article, authorship, publication outputs, and keyword frequency, using mathematical and statistical tools to describe, evaluate, and forecast the current state and trajectory of technological progress. CiteSpace, an acronym for “citation space,” is a citation visualization software specifically designed for scientific analysis and the discovery of underlying knowledge. It has been developed in the domains of scientometrics, data visualization, and information visualization [17]. CiteSpace offers three core bibliometric analysis functionalities: (i) Collaborative mapping, which assesses the academic impact of authors, institutions, and countries by examining their social connections within a particular domain (ii) Co-occurrence mapping, centered around keywords, investigating the forefront of research, hot topics, and their evolution, and (iii) Co-citation mapping, focusing on literature, excavating key documents in the research field and describing their interrelationships. The visualization results can be displayed in three distinct formats: cluster view, timeline view, and time zone view [18, 19].

The CiteSpace 6.3. R1 (64-bit) version was employed for data processing. Selected data sets from 2005 to 2024, including information for title, abstracts, author keywords (DE), and supplementary keywords (ID). The Node Types we analyzed include authors, countries, keywords, and references. We adjusted Years per Slice based on different Node Types and kept the default settings for other parameters. The COSINE algorithm was applied to determine link strength, and the minimum spanning tree and pruning slicing networks were combined. This article reviews the literature on the applications of chest LDCT in the diagnosis and treatment of lung diseases in the past two decades. Utilizing the theoretical framework of cluster analysis and the addressing network algorithm (PF-NET), keywords, authors, countries, and references are established as node types in the paper data. Then, the critical path method is used to analyze the data collection elements and construct the knowledge map. The co-occurrence map is utilized to investigate the research hotspots over the years, while the cluster view is used to reveal the development relationship between these hotspots. This study further analyzed the research trends in this field, aiming to provide a reference for the research of CT imaging technology and clinical diagnosis and treatment.

### 2.2 Data Sources

In this study, data are derived from the core collection of the Web of Science (WOS) database, including the Science Citation Index Expanded (SCI-EXPANDED), Social Sciences Citation Index (SSCI), Arts & Humanities Citation Index (A&HCI), and Emerging Sources Citation Index (ESCI). These databases jointly constitute the world's most extensive and comprehensive academic resource platform and offer the most extensive academic range. This study selected the topics of “low-dose” AND “CT imaging” AND “lung” for preliminary screening of articles and restricted the literature types to “articles” and “review articles”, with language selection limited to English. The search period is from 2005 to 2024, with a total of 2110 related articles, including 2055 research articles and 45 review articles. In order to reduce potential bias caused by frequent database updates, all literature searches and data downloads were conducted on July 1, 2024.

## 3 RESULTS

### 3.1 Analysis of the Number of Papers and Time Distribution

**Fig. (1):**
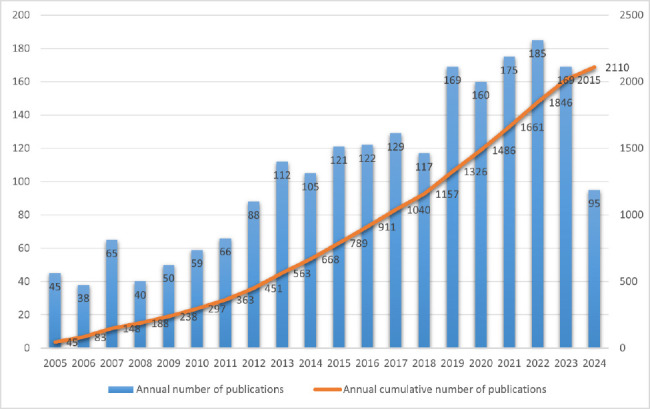
Statistics of the number of publications.

The annual publication volume is the main indicator of the feasibility of scientific research, and examining the temporal distribution of these publications allows us to determine changes in a field. In order to conduct in-depth research and examination on the application of LDCT technology in the field of lung disease, this study analyzed literature from the past two decades and drew a bar chart and a trend curve of the annual number of articles. The results indicate that the number of research papers in this field is considerable, showing an overall upward trend, and no period of research interruption (Fig. **1**).

From the annual cumulative number of publications, the overall trend showed an upward trend. Between 2005 and 2023, the number of annual publications present three distinct phases. From 2005 to 2011, the output remained relatively stable. Between 2012 and 2018, the number of publications increased steadily. By 2019, a significant surge in publications was observed, and the number of publications remained generally stable from 2019 to 2023. In 2005, despite limited progress, international interest in applying LDCT to the study of lung diseases began to escalate. There are two main reasons for this situation: firstly, the basic theoretical research and cognitive model in this field have not been fully developed in the world; secondly, the limitations of technical tools, including hardware, software, and data processing capabilities [20].

With the development of technology and in-depth theoretical research, the number of research results has increased, and the impact has become greater, which has attracted more attention. By 2019, the outbreak of SARS-CoV-2 had led to rapid development in research on lung diseases, especially in the mechanism of lung injury by SARS-CoV, the pathogenesis and treatment of Acute Respiratory Distress Syndrome (ARDS), and the lung complications associated with “long SARS-CoV-2” [21]. This study aims to use LDCT technology to improve treatment strategies, early detection, and continuous monitoring of lung diseases. In addition, research focuses on the development of new drugs and therapies to reduce lung damage and promote recovery. In 2023, the World Health Organization announced that COVID-19 would no longer be classified as a “Public Health Emergency of International Concern” (PHEIC), marking the stabilization of the epidemic's evolutionary stage. Therefore, the number of articles on LDCT and related lung research has increased during this period [22]. Ninety-five papers have already been published in the past six months, and it is expected that the annual total will exceed the peak of 2020 (185 papers), emphasizing the sustained interest in LDCT research.

### 3.2 Analysis of Issuing Countries

To analyze the output of countries that published literature between 2005 and June 2024, we split them into one-year increments and set a k value of 25. Therefore, it resulted in a co-occurrence map featuring 64 nodes, as shown in Fig. (**2**). In the figure, each node corresponds to a country, the lines connecting the nodes represent the cooperation between these countries, and the size of the nodes is directly proportional to the number of articles in each country [23]. The picture shows that nodes representing the United States, Germany, Italy, and Japan change from light to dark colors, while the node representing China displays stronger colors compared to other nodes. This indicates that although China later entered the field, its research output is still expanding rapidly. The United States not only has the largest nodes but has also many lines connected to it, indicating that they have made significant contributions in this research field and established cooperative relationships with many countries.

To gain insight into the nodal hierarchy of the field and to further dissect the academic output of LDCT techniques related to lung disease in different countries (Table **1**). We examined the number of publications. From this analytical perspective,, the United States, China, and Germany emerge as the leading countries with the highest number of research papers in this field, followed by Italy, the Netherlands, and Japan. Intermediate Centrality stands out as a pivotal metric in network analysis [23], quantifying the significance of nodes in the path connecting other nodes in the network. Nodes with higher centrality scores play vital role in the network and have more connections. Observing Table **1**, the United States exhibits the highest centrality at 0.31. The maturity of computer technology in the United States is the earliest globally, which shows that basic research in this area is largely dependent on American research results to borrow, expand, and innovate. Although Canada and England do not lead in the number of published papers, they are in the top three for centrality, indicating that these nations are closely linked to other countries in this research domain and hold significant positions.

### 3.3 Analysis of the Authors

When analyzing authors from 2005 to June 2024, we adopted a time slice of 3 and a k value of 15 to construct a knowledge graph with 296 nodes, as shown in Fig. (**3**). This study indicates that there are limited independent research teams in the application of LDCT in the field of lung diagnosis. Most research has been conducted through close collaboration between small groups. Table **2** lists authors who have published over 20 articles. This trend highlights the vibrant international cooperation and exchange in this field.

In the field of radiology, Dr. Vliegenthart Rozemarijn, a medical practitioner affiliated with the University of Groningen, was an early adopter of radiological techniques, CT, and other investigative avenues. Recently, she and her team have begun a thorough study on how uncertainty quantification affects the efficacy of Deep Learning (DL) algorithms in assessing the malignant potential of lung nodules [24]. They integrated the uncertainty estimation technique into the DL algorithm previously designed for assessing the risk of pulmonary nodule malignancy and evaluated its effectiveness. It was observed that the algorithm tended to classify larger benign nodules as uncertain, as well as some solid and non-solid malignant nodules. This suggests that these characteristics of nodules may be contributing factors to the algorithmic uncertainty. Oudkerk Matthijs, a professor of radiology at Leiden University in the Netherlands, specializes in radiology, lung diseases, and CT radiation doses. He summarized recent advances in the development of AI-based pulmonary nodule detection systems using the LUNA16 dataset. He noted that the AI system was significant at detecting solid nodules, identifying nodules even under LDCT scan conditions, although this may elevate the false positive rate of diagnosis. These insights will be invaluable in enhancing the AI performance in lung nodule detection [25]. Kauczor Hans-Ulrich from the University of Heidelberg specializes in medical imaging and lung disease analysis. His team validated that deep learning-based convolutional neural networks can predict lung cancer and demonstrated excellent performance on independent datasets from multi-center trials in Europe. This provides a solid foundation for clinical application and helps reduce unnecessary follow-up tests [26]. Furthermore, we observed that previous authors had collaborated on various projects, and the author co-occurrence map shows that research teams in this field are highly collaborative and that their research questions are closely interconnected.

### 3.4 Analysis of Highly Cited Literature

This section aims to analyze the extensively cited literature in the field of lung disease research, with an emphasis on LDCT technology. Considering the large number of articles referenced, Table **3** compiles the 15 most frequently cited studies. Early detection and treatment of lung disease, especially lung cancer, has attracted great attention. Dr. Aberle and her team found that LDCT is able to identify a range of tumors at an early stage, and the implementation of LDCT screening may help reduce lung cancer mortality rates [27]. De Koning HJ et al. explored whether volume CT screening could diminish lung cancer mortality among male smokers and those who quit before surgery, and the results showed that individuals who underwent volume CT screening had a significant reduction in lung cancer mortality compared to those who did not [28]. Bach PB et al. performed a systematic review of the advantages and disadvantages of LDCT screening for lung cancer, concluding that LDCT might be beneficial for individuals with a higher risk of lung cancer, although there are still uncertainties about the potential harms and the generality of the findings [29]. Krist AH and the US Preventive Services Task Force assessed the efficacy of LDCT and chest radiography in screening average or high-risk asymptomatic populations for lung cancer (including current or former smokers), and provided insights into the optimal age for starting screening, screening intervals, and the relative benefits and detriments of various screening methods through modeling studies [30]. The research indicated that LDCT screening positively affects the early treatment of lung cancer, and as the study progresses, it is anticipated that the potential shortcomings of screening will be mitigated, enhancing the efficacy of the screening process.

### 3.5 Analysis of Keywords

Keywords play a crucial role in academic papers, enabling researchers to understand the theme of the literature quickly. Analyzing keywords, can help classify literature and analyze research hotspots and emerging trends, as well as reveal the knowledge framework and research trajectory of academic research. It also provides new ideas for future research work and improves the productivity of academic work [31].

**Fig. (5):**
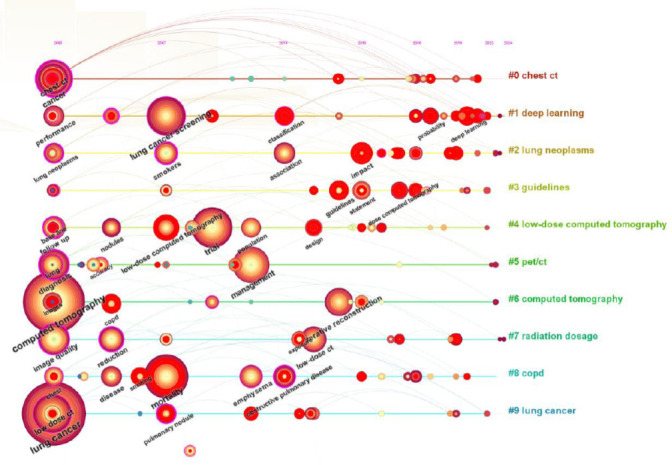
Time series clustering map of keywords

A map consists of 272 nodes, and they represent keywords, as shown in Fig. (**4**). A large number of lines between nodes indicate a strong correlation between them. The figure illustrates the extensive research on CT images and lung cancer, particularly in the fields of chest CT, lung nodule detection, and lung cancer diagnosis. With in-depth research, LDCT technology has assisted in the study of lung diseases. Due to the influence of radiation dose on CT imaging, researchers are increasingly concerned about the interaction between CT image quality and radiation dose. The quality of LDCT images has become a core topic of research. Our investigation also performed a cluster analysis on the sorted keywords (Fig. **5**), identifying the top 10 categories for the generation of the time series map. These categories can be categorized into three primary research domain, research subjects (#0 chest CT, #4 low-dose computed tomography, #5 PET/CT, #6 computed tomography), research inquiries (#2 lung neoplasms, #3 guidelines, #7 radiation dosage, #8 COPD, #9 lung cancer), and research methodologies (#1 deep learning). Early research mainly focused on research inquiries and subjects, followed by a shift in emphasis towards themes and methods. With ongoing technological advancements, recent research has centered on methodologies, utilizing new technologies to propose more effective solutions to the challenges identified in early and mid-term studies. CiteSpace assesses the network structure and the distinctiveness of clustering through module values (Q-values) and average silhouette widths (S-values). The Q value for this study stands at 0.8003, and the S value at 0.9275, signifying that the knowledge map generated by this study is compelling.

To present the focal points in the research domain more clearly and intuitively and to delve into the future trends of scholarly inquiry, this manuscript accentuates the significance of key terms. By pinpointing the most frequent and enduring keywords in the field, and integrating the years of these key terms, their most prominent years, and years of diminishing influence, we can track the evolution of the research landscape. Consequently, this survey demonstrates the research priorities and hotspots for a specific year and examines the trajectory of the field. The CiteSpace software was used to create a map featuring the top 25 keywords with the highlight value, ranked by the year they became salient, as depicted in Fig. (**6**).

## 4 DISCUSSION

### 4.1 Research Hotspot Analysis

Compared to previous bibliometric analysis studies [32], this study also used bibliometric methods for analysis. However, we analyzed the literature data on the application of CT technology in the diagnosis of lung diseases from different perspectives over the past two decades, mainly discussing three dimensions: research objects, research questions, and research methods.

The research initially focused on spiral CT technology and its use in early lung cancer screening. Subsequently, the scope was expanded to investigate the impact of radiation doses on human health. Eventually, the focus turned to solving noise problems in LDCT imaging. From 2005 to 2008, the spotlight was on the challenges of applying spiral CT technology, which became a central theme of research during that era. From 2009 to 2016, the research agenda was further expanded to include not only the exploration of lung diseases but also the interaction of these diseases with radiation exposure and the optimization of imaging techniques [33-35]. Between 2017 and 2024, with the popularity of chest LDCT, the research focus shifted to analyzing image quality, with a particular focus on noise reduction due to lower radiation doses.

With the deepening of research, the focus of the problem has changed. Initially, research focused on improving the diagnostic accuracy of spiral CT and PET scans. After that, the research center shifted to the potential health risks associated with radiation exposure. In the recent research phase, the researchers have begun to explore how artificial intelligence can solve the quality problem of LDCT scan image noise. Between 2005 and 2008, the discussion revolved around the application of CT technology, particularly spiral CT, in the screening and diagnosis of early-stage lung cancer [36]. Since 2006, PET has garnered the attention of researchers as an emerging technology, with a particular focus on its role in diagnosing lung cancer. From 2009 to 2016, radiation safety became the focus of research, especially Thin-section CT [37] and radiation exposure [38], which caused widespread attention and debate. The researchers are not only interested in disease detection but also in the potential harm that radiation can inflict on the human body, especially the health problems for heavy smokers [39]. Additionally, the mortality rate of lung diseases has increased. Between 2017 and 2024, LDCT imaging technology introduced a new research agenda on how to maintain a low radiation dose while resolving image noise issues to improve the accuracy of lung disease diagnosis. At the same time, with the continuous progress of computer technology, using deep learning and artificial intelligence to enhance the efficiency of LDCT disease diagnosis has become a significant research topic.

The research methods have evolved from traditional CT imaging to complex technologies that combine deep learning and artificial intelligence. This change emphasizes the combination of technological breakthroughs and research needs to accelerate the progress of early diagnosis and treatment of lung diseases. From 2005 to 2008, the focus was on calibrating instrumental parameters for spiral CT and PET scans [40]. Thin-slice CT technology was dominant between 2009 and 2016, and researchers were also delving into methods to minimize radiation exposure and devising strategies to mitigate the effects of radiation by enhancing CT imaging capabilities [41]. Since 2017, despite traditional image processing techniques such as filtered back projection [42], iterative reconstruction [43], and model-based iterative reconstruction [44] have been explored, these methods are still struggling to improve the accuracy of lung disease diagnosis due to the large number of images, the variability of imaging equipment, and the unpredictability of noise type and intensity. With the rapid advancement of computer technology, since 2020, deep learning and artificial intelligence have been extensively applied in the field of LDCT imaging, particularly in the domain of medical image classification and detection [45, 46]. Researchers have used these techniques to improve the accuracy and efficiency of diagnosing lung diseases. Therefore, the focus of research methods has gradually shifted towards combining deep learning with LDCT technology to overcome diagnostic challenges associated with lung diseases.

### 4.2 Research Conclusion

This survey used bibliometric techniques and CiteSpace software tools to conduct an in-depth visual analysis of the literature related to chest LDCT technology, i over the past two decades from the Web of Science core database. The study closely examined key indicators, including the number of annual publications, the geographical distribution of these publications in different countries, the contributions of authors, highly cited articles, and popular keywords. This comprehensive analysis aimed to reveal the current status, research focus, and emerging trends of the discipline. Based on these analyses, the following conclusions can be drawn.

Over the past two decades, the number of articles on lung diseases and LDCT has been increasing. During the COVID-19 outbreak in 2019, the number of papers in the field increased rapidly. In 2022, the number of articles in this field reached its highest. Although there was a slight drop in the number of papers in 2023, 95 papers were published in the first half of 2024, indicating that there is still huge research potential in the field.

In terms of research contribution by different countries, the United States is the earliest and most active participant, and its advanced technology and rich experimental data enable it to have close cooperation with other countries. Chinas research in this field is close behind, experiencing two significant research peaks in 2019 and 2022 While other countries are also playing a role in this area of research.

Based on the authors' analysis, research in this domain shows a strong global relevance. Although many small research groups exist, they have established an extensive network based on cooperation. This close collaboration greatly enhances the effectiveness of advancing research endeavors.

Reviewing highly cited literature has clarified that CT technology has been widely used to support disease diagnosis. Studies have shown that screening with LDCT technology can help reduce lung cancer mortality and improve patient outcomes. They also suggest that there is a need to improve screening programs and establish a standardized imaging technology framework. At the same time, the literature highlights the potential adverse effects that LDCT screening may have on individuals and warrants further study.

Keyword analysis shows that LDCT images are increasingly becoming a research hotspot, and deep learning has become the main method in the field of medical image processing and diagnostic methods. In addition, LDCT technology plays an important role in implementing lung cancer screening programs.

From the perspective of research trends and hotspots, using AI tools and deep learning methods to improve imaging quality before detecting and classifying lesion structures to help diagnose cancer early has become a core research topic and trend in this field. In the future, the optimization of artificial intelligence algorithms and the expansion of applications will provide strong technical support for the medical field and help diagnose various diseases.

### 4.3 Research Perspective

Based on our previous analysis of key areas and conclusions, we propose several predictions regarding the future trends of LDCT technology in lung disease research. We anticipate that in the coming period, research emphasis in this domain will continue to focus on how toe effectively implement chest LDCT imaging to assist in the diagnosis of lung diseases, along with related research methodologies. Our considerations mainly encompass the following aspects.

The balance between image quality and radiation dose is currently the focus of research. At present, the research mainly focuses on the processing of phantom data and simulated low-dose data, while the processing effect on real human data is still limited, and has not been fully integrated with clinical practice. Further research is essential to enhance and refine the deep learning reconstruction algorithm for LDCT images. The aim is to maintain high-quality reconstructions with minimal real human data, thereby ensuring superior imaging for clinical application. Meanwhile, considering that LDCT imaging is affected by different types of equipment, individual patient factors, and radiation dose, this can lead to complex noise. Therefore, future research will focus on accurately identifying image noise. This will enable the implementation of targeted noise reduction technologies for various types of noise. The study also aims to find the optimal balance between image quality and radiation exposure. The issue of LDCT imaging quality can be solved not only by processing images directly, but also by solving the inverse problem of partial differential equations. In the future, not only can we optimize current denoising algorithms, but we can also consider a mathematical perspective, using deep learning methods to solve inverse problems. These ideas are expected to greatly improve the image quality of LDCT and provide more effective solutions for clinical practice.

Artificial intelligence has made significant progress by training a large amount of medical image data. Deep learning algorithms have the ability to detect lung nodules with different characteristics and distinguish between benign and malignant lung nodules effectively [47].Additionally, LDCT image reconstruction technology and tuberculosis detection technology can be integrated to develop professional LDCT image processing artificial intelligence software. This software aims to integrate with clinical practice to achieve real-time low-dose image processing and examination. It can be directly connected to imaging devices to provide accurate and intelligent diagnostic support for radiology. In addition, it can automatically identify and classify lung nodules, quantitatively analyze nodule features, accurately assess risks, and provide diagnostic reports and treatment recommendations after processing images. Lastly, the new software with AI technology promises to dynamically track the condition of lung nodules and provide disease alerts based on the individual patient's situation. The integration of this technology and the development of software will improve the effectiveness of early screening for lung cancer, helping to detect and treat major lung diseases early and improve survival rates. Simultaneously, postoperative physical condition can also be monitored to reduce the occurrence of other risks caused by diseases.

The integration of different medical imaging technologies plays a crucial role in evaluating the degree of lung cancer infiltration and surrounding tissue structure [48]. It can obtain high-resolution anatomical images as well as metabolic and functional data to comprehensively evaluate the structure of lung lesions. The development of multi-modal image fusion technology is expected to achieve real-time data acquisition and recognition of typical lesion features, aiming to achieve high-precision automatic diagnosis of basic lesion conditions. By integrating the physiological characteristics and genetic information of patients, disease analysis of different individuals can be achieved, which is helpful for accurate diagnosis and treatment. In addition, the platform hopes to utilize cloud computing and data-driven approaches to access global image databases, support multi angle comparative analysis, promote remote diagnosis, and significantly improve the diagnostic capabilities of medical institutions. Multi-modal image fusion ultimately aims to achieve intelligent services from initial disease screening to long-term health monitoring after treatment. This will promote disease prevention and health management effectively, and improve the public's health and overall treatment level comprehensively.

### 4.4 Study Limitations

This study has several limitations. Firstly, this research only included English-language articles and reviews from the Web of Science Core Collection, which may have excluded relevant studies from other databases (such as Scopus, PubMed) or unindexed publications, as well as non-English literature and under representation of regional journals, which means that we may have missed some valuable studies. Nevertheless, given the high coverage of WoS for the vast majority of research, we believe such omissions will not significantly impact the overall trends. Secondly, the scope of analysis is limited to publications within a set time period, which may not cover the latest progress. Additionally, due to the lag in citation impact, some recently published high-quality studies may have been underestimated in terms of their influence, which requires continuous follow-up and updates in subsequent research. Furthermore, the bibliometric analysis primarily focused on citation-based trend analysis rather than qualitative insights (such as theoretical depth or methodological rigor). Despite these limitations, this study will still provide valuable reference for the academic community to understand the development trends and cutting-edge hotspots of low-dose chest CT in the diagnosis of lung diseases.

## CONCLUSION

This study analyzed the relevant literature on the application of low-dose CT technology in lung diseases in the past 20 years through bibliometric methods. The research hotspots in this field focus on low-dose, radiation dose, image quality, and deep learning and so on. Combined with this analysis, we further discussed the future research trends in this field. In the end, the conclusion is as follows: Future research will focus on LDCT reconstruction algorithms to balance image noise and radiation dose. By deep learning and inverse problem methods, obtaining better results on small real data samples can enhance clinical value. AI-assisted multimodal imaging supports remote diagnosis and personalized health management, providing dynamic analysis, risk assessment, and follow-up recommendations to support early diagnosis.

## SUMMARY

This study only includes the WoS database and may not fully include English language publications, but a large amount of literature research also indicates that low-dose CT plays a key role in the diagnosis of lung diseases, especially in the early detection of lung cancer. With the continuous advancement of technology and the deepening of interdisciplinary research, we hope that the accuracy and sensitivity of early diagnosis of lung cancer will be greatly improved. This will markedly improve the efficacy of lung cancer prevention strategies and elevate the quality of life for patients. In the future, this area of research has great potential for growth in terms of technological innovation, clinical application, data analysis and policy support.

## Figures and Tables

**Fig. (2):**
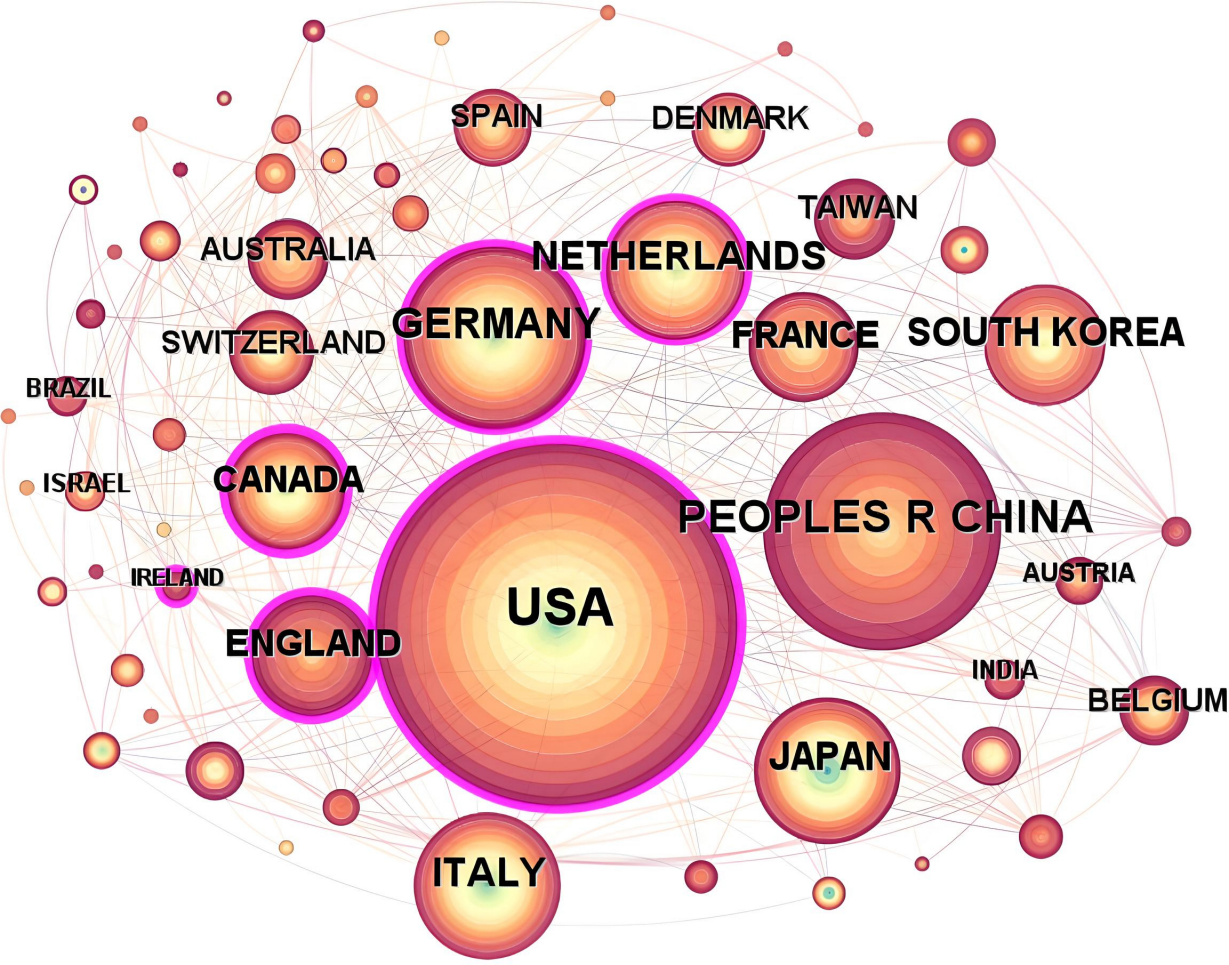
Co-occurrence mapping of issuing countries.

**Fig. (3):**
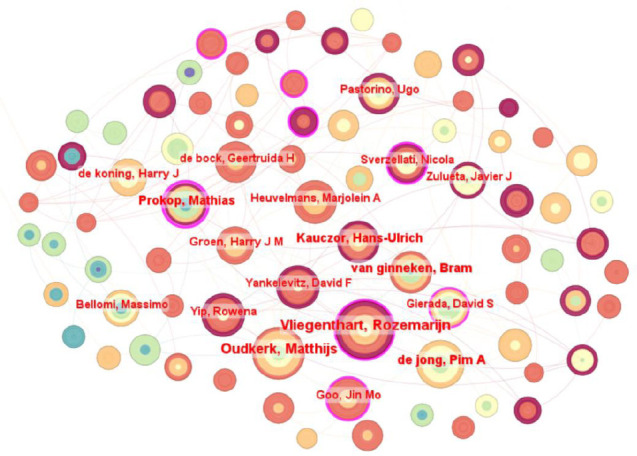
Co-occurrence mapping of issuing authors.

**Fig. (4):**
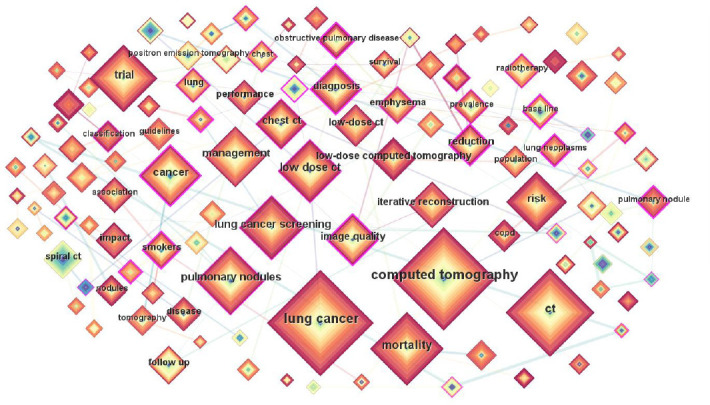
Co-occurrence network map of keywords.

**Fig. (6):**
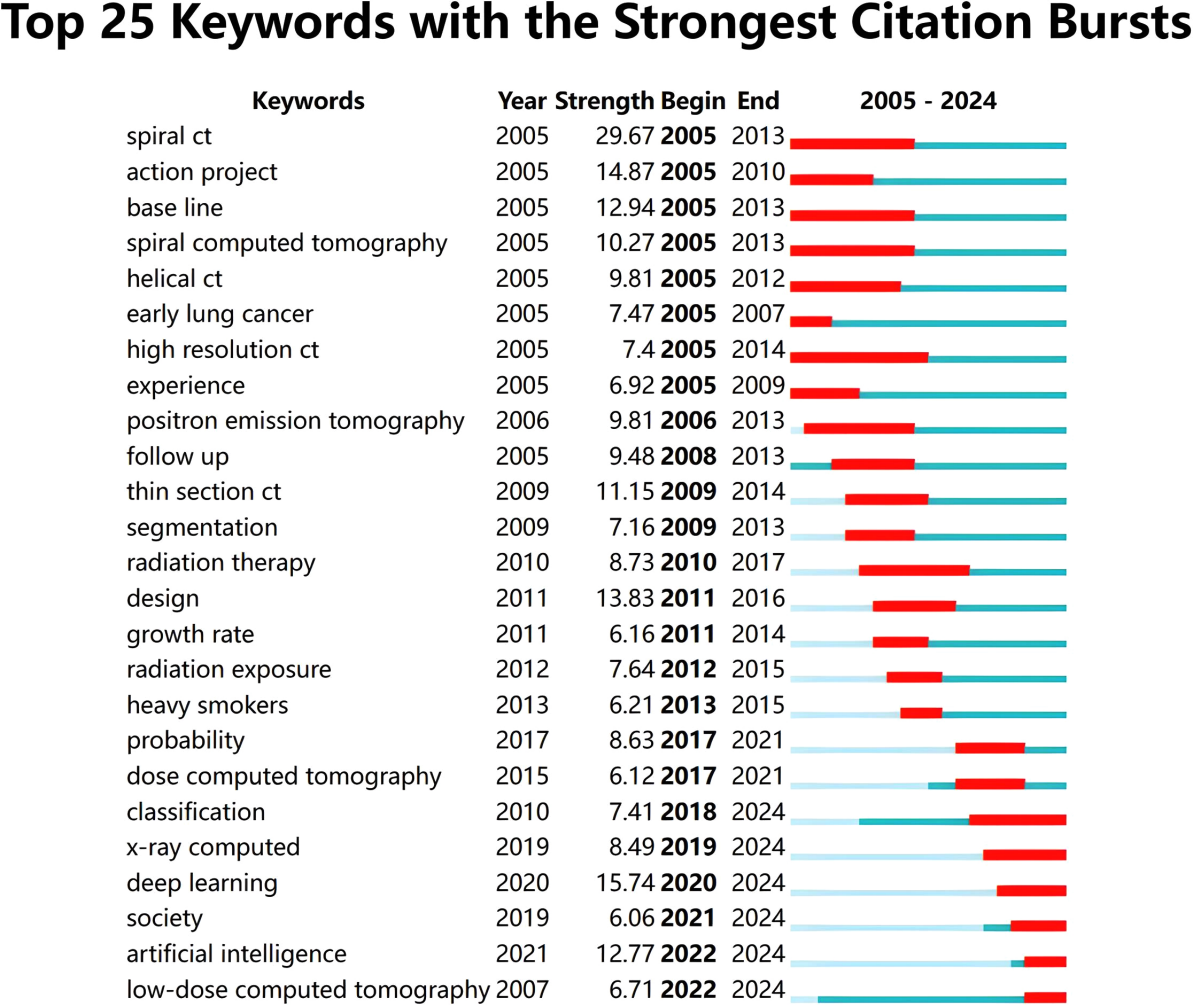
Highly prominent keyword map.

**Table 1:** The number of articles and the degree of the center ranked top 10, respectively.

Rake	Country	Amount	Country	Centrality
1	USA	733	USA	0.31
2	Peoples R China	333	England	0.26
3	Germany	229	Canada	0.25
4	Italy	161	Germany	0.16
5	Netherlands	150	Netherlands	0.11
6	Japan	138	Ireland	0.11
7	South Korea	119	Italy	0.09
8	Canada	108	Spain	0.09
9	England	101	Greece	0.08
10	France	91	Scotland	0.08

**Table 2:** The number of authors' publications ranked top 9.

Authors	The Initial Year of Publication	Number of Documents
Vliegenthart, Rozemarijn	2011	41
Oudkerk, Matthijs	2008	38
Kauczor, Hans-Ulrich	2008	25
De Jong, Pim A	2011	25
Prokop, Mathias	2006	24
Van Ginneken, Bram	2008	24
Goo, Jin Mo	2011	23
Groen, Harry J M	2012	22
de Koning, Harry J	2011	21

**Table 3:** Top 10 cited articles.

Author	Times Cited	Years	Document
Aberle DR	266	2011	Reduced lung-cancer mortality with low-dose computed tomographic screening
de Koning HJ	216	2020	Reduced Lung-Cancer Mortality with Volume CT Screening in a Randomized Trial,
MacMahon H	100	2017	Guidelines for Management of Incidental Pulmonary. Nodules Detected on CT Images: From the Fleischner Society 2017
Bach PB	91	2012	Benefits and harms of CT screening for lung cancer: a systematic review
Gatsonis CA	73	2011	The National Lung Screening Trial: Overview and Study Design
Krist AH	70	2021	Screening for Lung Cancer: US Preventive Services Task Force Recommendation Statement
Patz EF	70	2014	Over-diagnosis in low-dose computed tomography screening for lung cancer
Saghir Z	70	2012	CT screening for lung cancer brings forward early disease. The randomised Danish Lung Cancer Screening Trial: status after five annual screening rounds with low-dose CT
Van Klaveren RJ	63	2009	Management of Lung Nodules Detected by Volume CT Scanning
Pastorino U	58	2019	Prolonged lung cancer screening reduced 10-year mortality in the MILD trial: new confirmation of lung cancer screening efficacy

## Data Availability

The data and supportive information are available within the article.

## LIST OF ABBREVIATIONS

LDCT: Low-Dose Computed Tomography
CT: Computed Tomography
WOS: Web of Science
SSCI: Social Sciences Citation Index
ESCI: Emerging Sources Citation Index
ARDS: Acute Respiratory Distress Syndrome
PHEIC: Public Health Emergency of International Concern
DL: Deep Learning
